# Robot-assisted percutaneous pars–pedicle screw fixation for treating Hangman’s fracture

**DOI:** 10.1186/s13018-023-03765-x

**Published:** 2023-04-03

**Authors:** Jingwei Zhao, Yajun Liu, Qi Zhang, Mingxing Fan, Xiaoguang Han, Da He, Bo Liu, Wei Tian

**Affiliations:** 1 Department of Spine Surgery, Beijing Jishuitan Hospital (4Th Clinical Medical College of Peking University), 31 Xinjiekou East Street, Xicheng District, Beijing, 100035 China; 2 Research Unit of Intelligent Orthopedics, Chinese Academy of Medical Sciences, Beijing, China

**Keywords:** Robot-assisted orthopedic surgery, Spinal surgery, Minimally invasive surgery, Hangman fracture

## Abstract

**Background:**

This study aimed to evaluate the safety and efficacy of robot-assisted percutaneous pars–pedicle screw fixation surgery for treating Hangman’s fracture.

**Methods:**

The study involved 33 patients with Hangman’s fracture who underwent robot-assisted fixation surgery using cannulated pars–pedicle screws through a percutaneous approach. The primary parameter evaluated was the accuracy of the screws according to the Gertzbein–Robbins scale, using postoperative CT images. Secondary parameters included the duration of surgery, intraoperative blood loss, postoperative hospital stay, and neurovascular injury.

**Results:**

A total of 60 pars–pedicle screws were placed in 33 patients. Based on the Levine and Edwards classification, the patients included 12 cases of type I, 15 cases of type II, five cases of type IIa, and one atypical case. The average operative time was 92.4 ± 37.4 min, and the average blood loss was 22.4 ± 17.9 ml. Fifty-five of 60 screws were successfully placed within the bone. No screw-related neurovascular injury was observed, and satisfactory reduction was achieved in all cases.

**Conclusion:**

Robot-assisted percutaneous pars–pedicle screw fixation is a safe and feasible method for treating Hangman’s fracture.

*Trial registration*: The study was retrospectively registered and approved by our center’s institutional review board.

## Background

Hangman's fracture, also known as traumatic spondylolisthesis of the axis, is typically caused by hyperextension and axial loading with or without flexion resulting from incidents such as road traffic accidents, trauma, and falls. The Levine and Edwards classification system [1] categorizes fractures with displacement or angulation of C2 on C3 (types II, IIA, and III) as unstable and requiring rigid immobilization.

The treatment options for Hangman’s fracture usually are halo vest immobilization or surgery. The halo vest is traumatic and the patient suffers when required to immobilize for 12 weeks. However, surgery is often avoided due to the high risk of neurovascular injury. Inter-segmental fixation, typically C2–C3 fixation, decreases mobility. C2 pars–pedicle screw can achieve fixation across fracture line thus preserving inter-segmental motion. Several studies have described favorable clinical outcomes [2, 3]. This technique can benefit patients by enabling early rehabilitation and avoiding the need for unbearable halo vest immobilization [4]. However, the technique in previous reports required exposure to the posterior surface of the C2 vertebra.

Robot-assisted spinal surgery has shown to be highly accurate in both thoracolumbar and cervical surgeries [5, 6]. Also, it can facilitate minimally invasive surgeries (MIS) [7]. This study investigated the safety and accuracy of robot-assisted pars–pedicle screw fixation through a percutaneous approach for Hangman’s fracture.

## Methods

### Aim

To evaluate the safety and efficacy of robot-assisted percutaneous pars–pedicle screw fixation surgery for treating Hangman’s fracture.

### Study design

Retrospective case series. The study involved 33 consecutive patients with Hangman’s fracture who underwent robot-assisted percutaneous pars–pedicle screw fixation surgery in our center from Jan. 2016 to Jul. 2022.

### Surgical decision and postoperative management

**Fig. 1:**
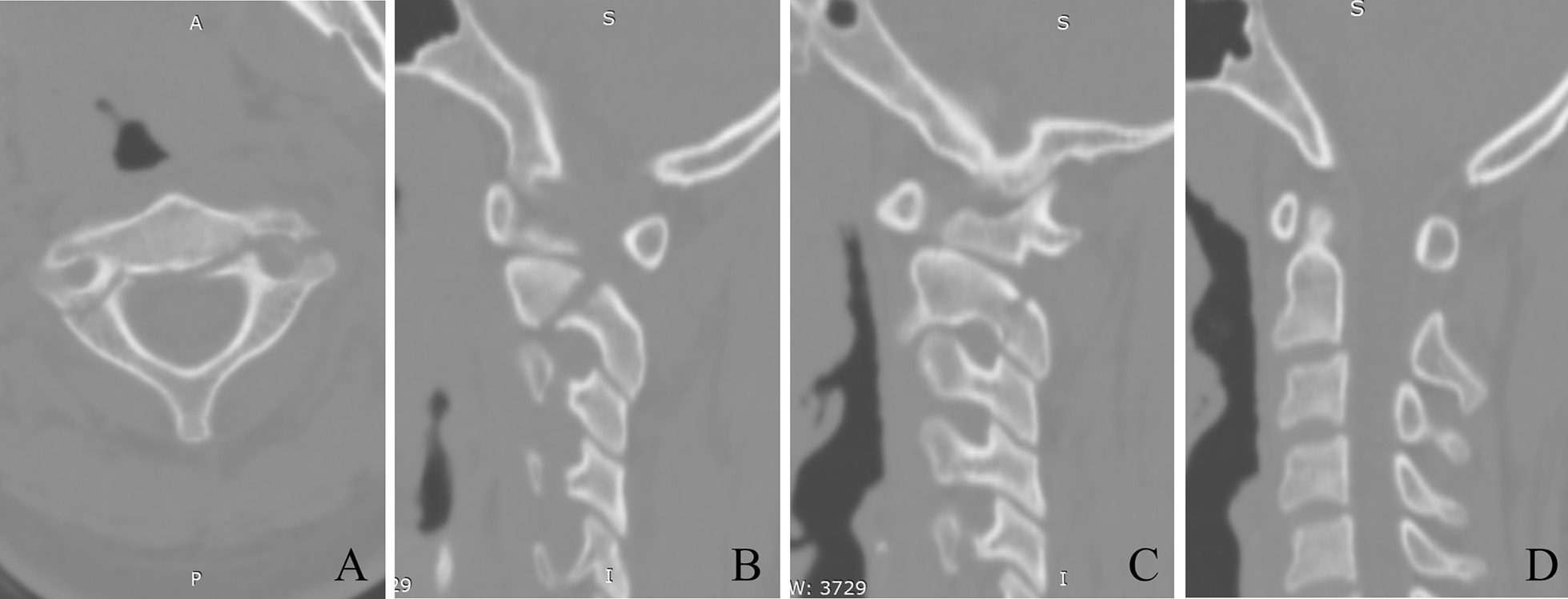
A typical patient. 19 y/o, female. Fracture lines are shown in **A** axial, **B** sagittal left pars, and **C** sagittal right pars. Type II was diagnosed by the translation of C2 on C3 for 3 mm (**D**)

The stability the pars–pedicle screw can provide is controversial, so the indication for the surgical method was carefully chosen. Patients with type I and type II with minimal translation (C2 on C3 less than 4 mm) and without severe disk disruption on MRI were considered for the surgery. (Fig. 1).

### Robot component

**Fig. 2:**
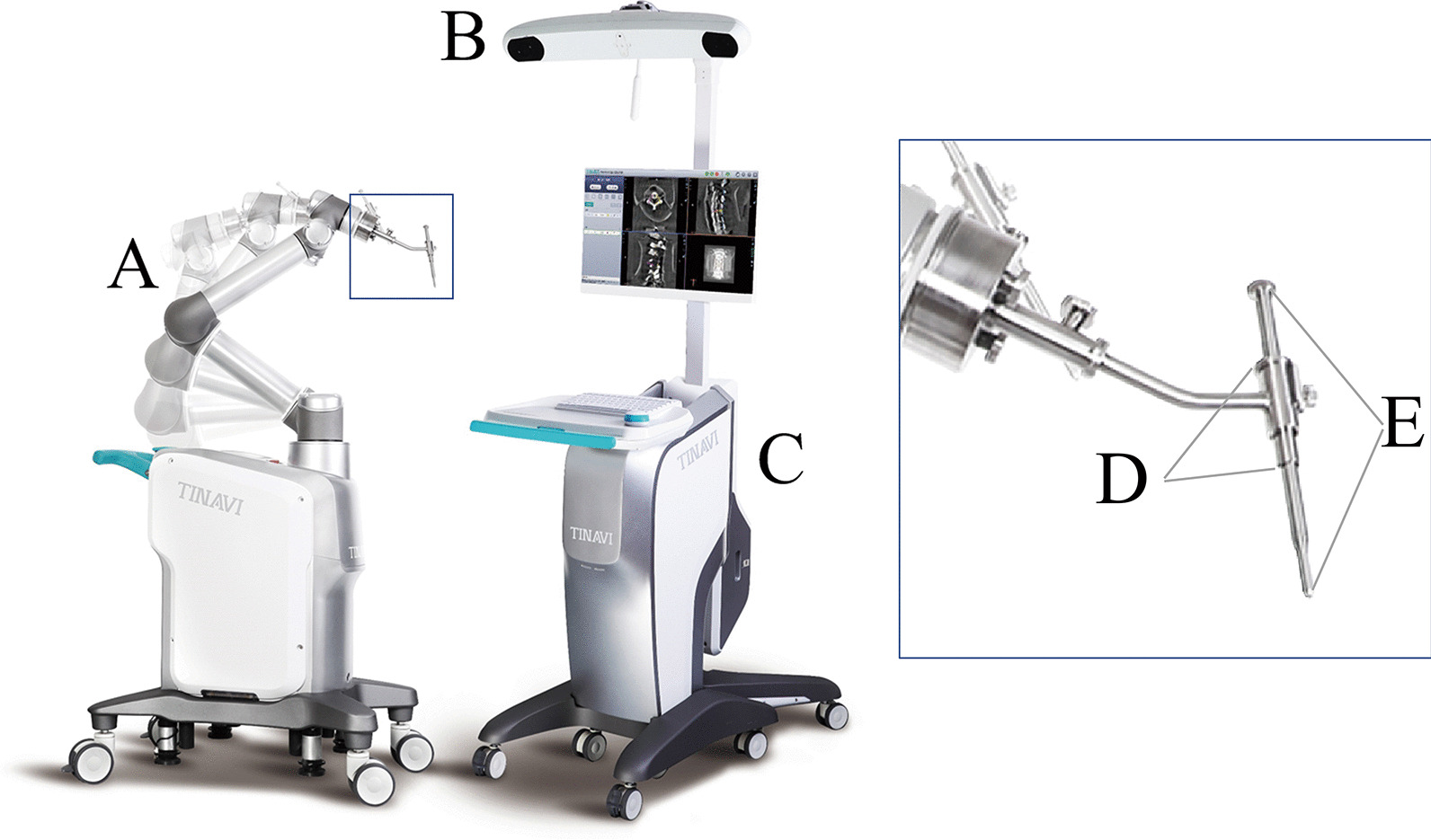
Tirobot system set. The system consists of **A** a 6-DOF robotic arm, **B** an optical tracking camera, and **C** a workstation. Zoomed image shows **D** the guider and **E** the cannulated guiding tube

The Tirobot system (TINAVI, China) comprises of an optical tracking camera, a surgical planning and controlling workstation, and a robotic arm. The binocular tracking camera locates the spatial positions of the patient and the robotic arm via trackers. The 6-degree-of-freedom arm is equipped with a guiding tube that allows for accurate positioning and guidance under the control of the tracking device and workstation. (Fig. 2).

### Surgical procedure

**Fig. 3:**
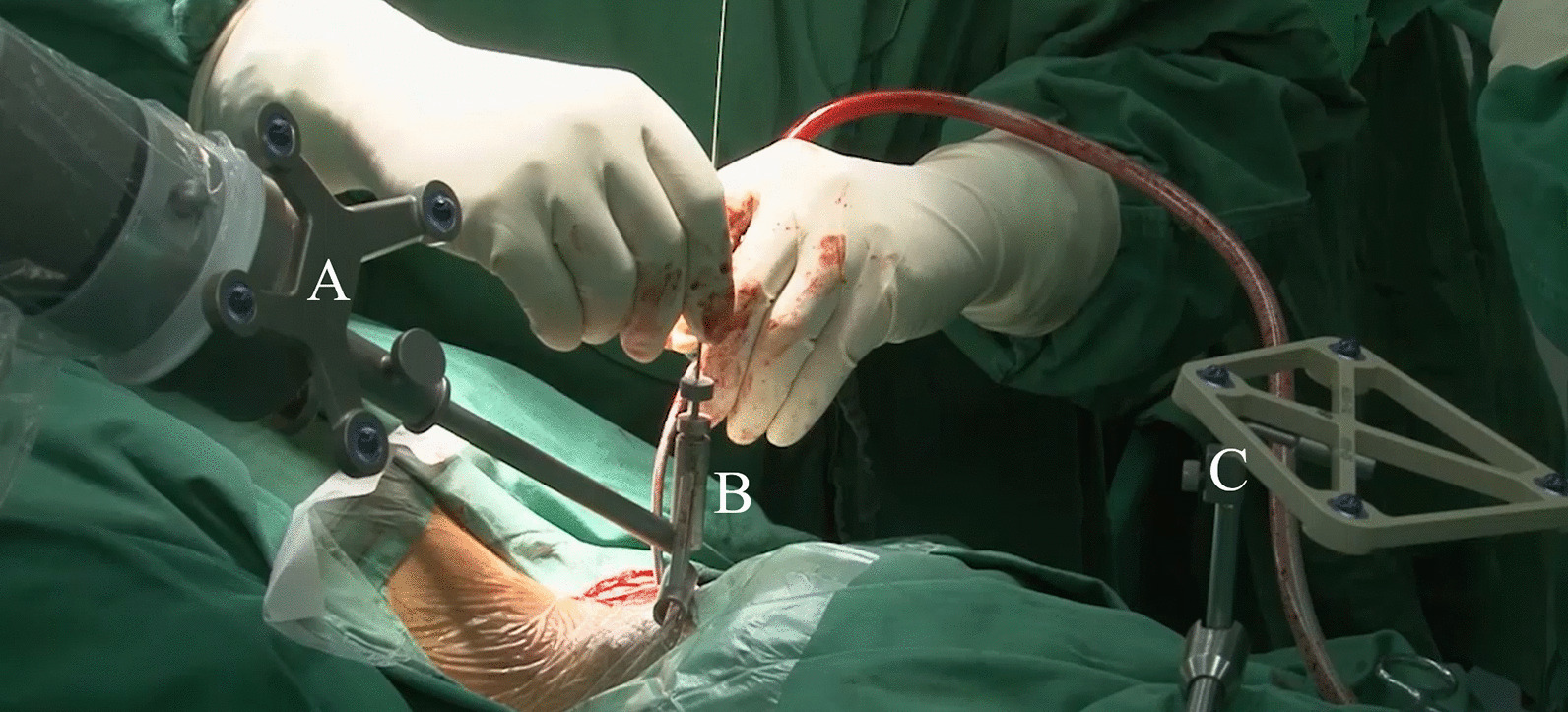
Instrumentation. **A** The robot arm with a tracker; **B** The guider with the guiding tube inside; **C** The patient tracker attached to the Mayfield frame

(1) Patient positioning. The patient was positioned prone on a radiolucent table with the head immobilized to the table using a Mayfield frame. If translation was found between the fragments, we would apply axial traction and reduction by moving the Mayfield frame cephalically and posteriorly until lateral X-ray showed satisfying reduction. (2) Patient tracker fixation. The patient tracker was fixed onto the Mayfield frame as accurate navigation relies on a rigid connection between the patient tracker, frame, and head. Spinous processes are commonly used as anchor points for trackers in open surgery, while they are not exposed in minimally invasive surgery. (3) Image acquisition. Intraoperative computed tomography (CT) was acquired using an ISO-C arm scanner (Siemens, Germany). The CT images was then transferred to the robot system, and spatial registration was automatically done. Thus, the robot camera can locate and trace the positions of the patient and the guider. 4) Screw planning. On the robot workstation, the pars–pedicle screws across the fracture line were manually planned based on all three reconstructed multiplanes of the CT: axial, sagittal, and coronal. (5) Robot arm positioning. The robot arm with the guider then steered itself toward the chosen trajectory. This process was automatically done by the robot system. After the positioning process, the axis of the guider and the planned screw would be right aligned. (6) Minimal skin incision. A guiding tube was inserted along the guider. The skin and fascia incision were accurately indicated by the guiding tube inside the guider. The length of the incision should be limited to fit the screw insertion without extra exposure. Then, the guiding tube, which was coaxial with the guider, was inserted onto the C2 lamina. (7) Guiding wire insertion. Through the guiding tube, the guiding wire was inserted into the bone using an electric drill. (Fig. 3) This step was double-checked using lateral and anteroposterior X-ray images during the insertion. An intraoperative CT scan was performed to confirm the position of the guiding wires. (8) Cannulated screw insertion. Tap threads were made using a cannulated tap and 4-mm-diameter cannulated screws were inserted along the guide wires.

### Postoperative management

Postoperatively, patients were immobilized using Philadelphia collar for 8–12 weeks. Patients were allowed to ambulate on the first day after surgery. Postoperative CT scans were performed for all patients before discharge.

### Screw accuracy measurement

The accuracy of the screw placement was evaluated using postoperative CT by one experienced surgeon. According to the Gertzbein and Robbins scale, the screw position was classified into three grades: A (screw completely within the pedicle), B (pedicle cortical breach less than 2 mm), and C (pedicle cortical breach no less than 2 mm). The breached direction of the pedicle was also recorded.

### Secondary parameters

The secondary parameters include duration of surgery, intraoperative blood loss, postoperative hospital stay, and neurovascular injury. Patient characteristics including sex and age were also recorded.

### Statistical analysis

All statistical analyses were performed using SPSS 25.0 (IBM, USA). Values are presented as mean ± standard deviation.

## Results

A total of 33 patients were included in the study. All surgeries were successfully performed through a percutaneous approach. There were 18 males and 15 females. The mean age was 42.8 ± 13.8 years. The fractures were classified with 12 type I, 15 type II, five type IIA and one atypical. The atypical one combined a unilateral pars fracture with a dens fracture. Sixty pars screws were instrumented. Twenty-seven patients were bilaterally fixed and six were unilaterally fixed.

**Fig. 4:**
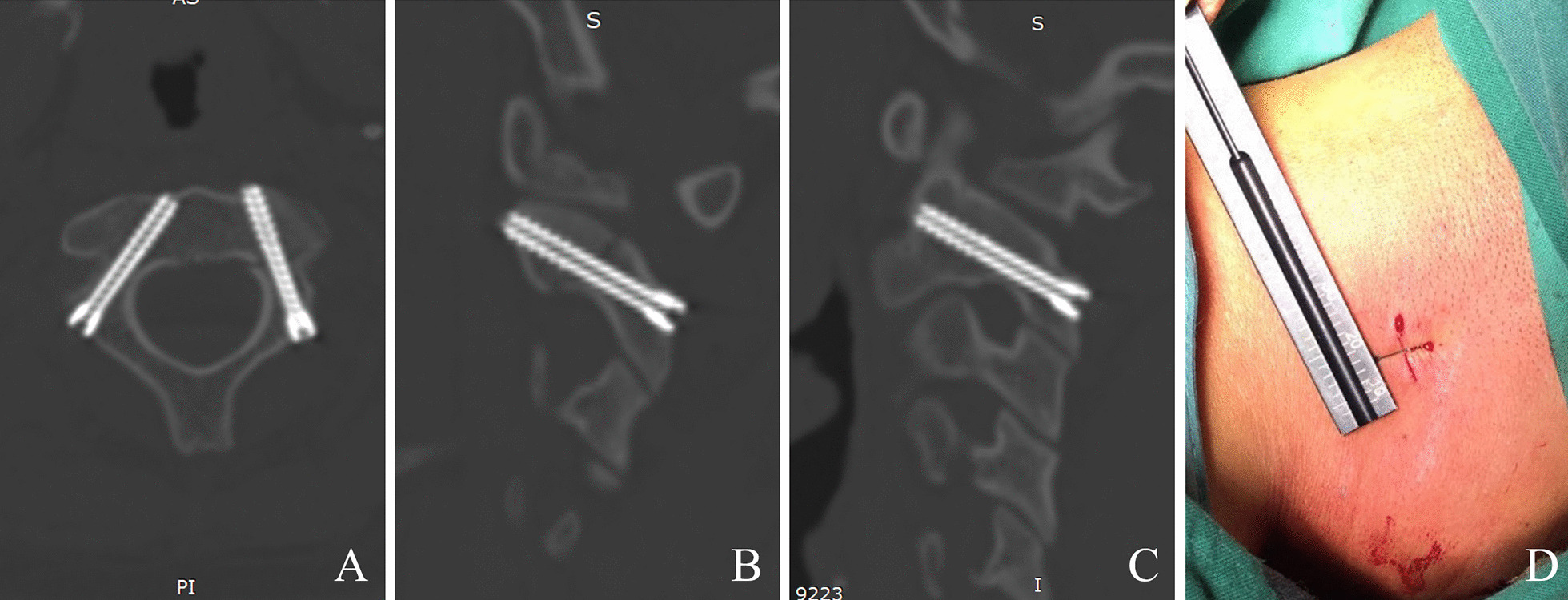
A typical patient after surgery. Pars screws are shown in **A** axial, **B** sagittal left pars, and **C** sagittal right pars. The length of each skin incision was 1 cm (**D**)

For the primary parameter, 91.7% of the 60 screws were perfectly placed (grade A), among which 48 screws did not violate the cortical of pars, pedicle, or transverse foramen assessed based on postoperative CT scan, and seven screws violated but did not breach the cortical of the transverse foramen. 8.3% of the screws breach the cortical of the transverse foramen for less than 2 mm (grade B). No neurovascular injury was observed during surgery or after surgery. (Fig. 4).

Six patients (out of 33) underwent unilateral screw fixation. In three cases, the fracture lines on the contralateral side were through the lamina. In two cases, the pars were too thin (less than 2 mm) to implant. And in one case, the pars fracture was unilateral combined with dens fracture and a dens screw was implanted through a previous robot-assisted anterior approach.

For secondary parameters, the average surgical time was 92.4 ± 37.4 min. The average blood loss was 22.4 ± 17.9 ml. The average hospital stay after surgery was 2.6 ± 1.0 days.

## Discussion

Pars screw fixation through a minimally invasive approach under the guidance of 3D navigation has been reported [3, 8]. In our experience, robot-assisted surgery has the following advantages: (1) During robot-assisted surgery, the guiding wire is placed by a drill, which may reduce the force onto the bone and improves accuracy. In navigation-assisted surgery, the probing force “pushes” the bone away, which will cause relative movement between the target bone and the patient tracker, resulting in accuracy deterioration. This is more notable in cervical bones since the flexibility is high but the fault tolerance is low. In comparison, guiding wire drilling causes less movement of the bone during robot-assisted surgery. (2) The skin incision is reduced to 1 cm (Fig. 4) per screw, compared with the reported 4 cm MIS approach [4]. Previously reported MIS approaches, whether under fluoroscopy or navigation, must require visual exposure of the entry point on the bone. However, robot-assisted surgery avoids visual exposure of the entry point by using the guiding tube. The tip of the guiding tube is positioned onto the entry point under the guidance of the robotic arm. Through the guiding tube, the guiding wire can be accurately placed.

Caution should be taken when doing robot-assisted upper cervical surgery. (1) Place the trackers firmly onto the patient or the robotic arm. The Mayfield frame is a good device to attach the tracker onto because the junctions are rigid and it facilitates minimal incision as shown in this study. (2) Make the screw plan carefully because it will be executed accurately and there will be little chance to manually modify it during the guiding wire insertion. The screw should be as perpendicular as possible to the fracture line to achieve good reduction and fixation, while the bone surface of the entry point should be not too steep to prevent wire slipping. The vertebral artery, which is commonly variated, should also be fully dodged. These requirements are possible thanks to the planning based on multiplanar reconstruction along/perpendicular to the screw of the intraoperative CT.

Twelve screws violated the cortical bone of the transverse foramen, among which seven did not breach and five breached for less than 1 mm. The violated parts of the C2 transverse foramen were all cephalic and medial, where the risk of vertebral artery injury is relatively low. In comparison, freehand C2 screw instrumentation may cause 11–23% cortical breach, among which 13% breach was over half of the screw diameter over the cortical edge [9]. An anatomic study showed that the injury rate of the vertebral artery groove of C2 caused by anatomic variations could be as high as 8% for C2 screw instrumentation [10]. Robot-assisted surgery may reduce the rate of the cortical breach, especially severe breach, thus reducing the risk of neurovascular injury.

The indication of the C2 pars–pedicle screw fixation for hangman fracture is controversial. Pars–pedicle screw fixation can restore the stability of lateral bending and axial rotation to a nearly normal state and reduce the instability of flexion and extension [11]. Another study shows that pars–pedicle screw may be not suitable for translation injury over 4 mm [12]. In this study, we chose patients with type I and type II with minimal translation to undergo the surgery. After the surgery, patients were required to immobilize under a Philadelphia collar in compensation for the remaining instability of flexion and extension.

Radiation exposure affects the health of the patients and surgeons. In this study, two intraoperative CTs and one postoperative CT were performed in each case, which may contribute to higher radiation dose to the patients. However, robot-assisted technique may result in decreased radiation exposure to the surgeons compared with that in the conventional technique, since the surgeons would leave the operating room during the CT imaging [5].There are limitations in this study. The study consists of only 33 patients because the indication was carefully chosen for the technique as previously described. The study consists of no control group of freehand or navigation-assisted surgery. Since the computer-assisted navigation technique was introduced to our center in the early 2000s, there were few freehand upper cervical surgeries. Navigation-assisted pars–pedicle screw fixation surgery was rare because the iatrogenic trauma from the approach is considerable therefore conservative treatment, usually, a halo vest would be chosen for similar patients as in this study before the robot-assisted percutaneous technique was available.

## Conclusions

Robot-assisted pars–pedicle screw fixation through a percutaneous approach is proved to be safe and feasible for treating Hangman fractures.

## Data Availability

The datasets analyzed during the current study are available from the corresponding author on reasonable request.

## Abbreviations

MIS: Minimally invasive surgeries
CT: Computed tomography
